# Differential Expression of MicroRNAs in Papillary Thyroid Carcinoma and Their Role in Racial Disparity

**DOI:** 10.4172/1948-5956.1000340

**Published:** 2015-05-25

**Authors:** Raagini Suresh, Seema Sethi, Shadan Ali, Tamar Giorgadze, Fazlul H. Sarkar

**Affiliations:** 1 Department of Pathology, Wayne State University School of Medicine, Detroit, Michigan, USA; 2 Oncology, Karmanos Cancer Institute, Wayne State University School of Medicine, Detroit, Michigan, USA; 3 Weill Cornell Medical College-Cornell University, New York, New York, USA

**Keywords:** miRNAs, Papillary thyroid cancer, Racial disparity, qRT-PCR, Targeted therapies, FFPE

## Abstract

**Objective:**

MicroRNAs (miRNAs) are known to play important roles in the diagnosis and prognosis of papillary thyroid cancer (PTC), and they are useful in developing targeted therapies. However, there have been no studies on the existence of racial differences in miRNAs expression that could explain differential overall survival of PTC patients. Expression analysis of miRNAs in major racial groups would be important for optimizing personalized treatment strategies. In the current study, we assessed the differential expression of 8 miRNAs between normal and tumor tissues, and also assessed racial differences between African American (AA) and Caucasian American (CA).

**Methods:**

First, the miRNA expression profiling was performed using formalin-fixed paraffin embedded (FFPE) tissue sections of tumor containing over 70% tumor cells. Normal and tumor sections of thyroid tissues were studied from AA and CA patients. The miRNA microarray profiling was done using miRBase version 18 (LC Sciences, Houston, TX, USA). Quantitative real-time PCR (qRT-PCR) was used to validate expression of 8 selected miRNAs.

**Results:**

Ingenuity pathway analysis showed involvement of target genes, such as Ras and NF-κB. Deregulated miRNAs such as *miR-221* and *miR-31* were found to be statistically significant between the two races. Using qRT-PCR, we found that *miR-21*, *miR-146b*, *miR-221*, *miR-222*, *miR-31*, and *miR-3613* were up-regulated while *miR-138* and *miR-98* were down-regulated in tumors compared to normal tissues.

**Conclusion:**

Though sample size was small, we found several deregulated miRNAs having racial differences. The differential expression of miRNAs suggest that these miRNAs and their target genes could be useful to gain further mechanistic insight of PTC and their clinical implications, including miRNA replacement therapy or their knockdown strategies.

## Introduction

Papillary thyroid carcinoma (PTC) is the most common form of thyroid cancer, comprising about 85% of all thyroid cancer [1]. Though its mortality rate is relatively low, its increasing incidence begs further investigation into the molecular basis of PTC [1,2]. In this regard, a promising area of study is to ascertain differential expression of microRNAs (miRNAs), which are known to function as key regulators of gene expression.

The miRNAs are small sequences of non-coding RNA which plays key roles in posttranscriptional gene regulation. They affect gene expression by base pairing with complementary sequences, usually at the 3′-UTR of target mRNAs, and affecting target protein levels [3]. There are two primary methods in which miRNAs could contribute to cancer development. For one, the overexpression of certain miRNAs could lead to the repression of tumor suppressor genes. Alternatively, miRNA down-regulation could result in increased oncogene expression [4]. The ultimate consequence is the onset of tumorigenesis due to the loss of expression of tumor suppressor genes and/or over expression of oncogenes.

The aberrant expression of miRNAs has been observed in various types of human cancers, including but not limited to PTC [5]. Previous studies sought to determine which miRNAs are differentially expressed in PTC as opposed to normal tissue. Many studies have determined that there are heightened expressions of *miR-21*, *miR-146b*, *miR-221*, *miR-222*, and *miR-31* in PTC [3,6–14]. The *miR-138* has been less extensively studied, but one study performed miRNA array that identified *miR-138* down-regulation in aggressive PTC compared to nonaggressive PTC [10]. Similarly, *miR-98* has been previously found to be down-regulated in PTC [15].

One aspect of PTC that has yet to be examined is the differential expression of miRNAs among the two major racial groups such as Caucasian American (CA) and African American (AA). Prior findings have demonstrated that AA or being a minority racial group is a significant risk factor for overall survival when considering PTC tumors of size 1cm or less [16]. In previous studies, it has also been shown that the incidence of thyroid cancer in AA is half that of CA [17]. However, the recent pilot study from our group that analyzed the AA population with respect to fine needle aspiration of thyroid lesions in correlation with surgical pathology follow-up showed similar distribution of benign vs malignant lesions in both groups of patients [18]. These observations suggest that there is a critical need for further elucidation on the differential expression of miRNAs in PTC among AA and CA patients’ specimens.

Therefore, our current study was focused on the evaluation of miRNA expression profiles using FFPE samples from CA patients (n=14 for 3 miRNAs, n=13 for the remaining 5 miRNAs due to insufficient size of tissue samples) and from AA patients (n=8 for 3 miRNAs, n=5 in the remaining 5 miRNAs due to the similar sample limitation). Two samples were obtained from the thyroids of each patient studied, one from normal tissue and one from malignant tissue. Following the evaluation of miRNA expression profiles, further validation of selected miRNAs was performed using quantitative real-time PCR (qRT-PCR) of individual samples. Here we report the results of miRNAs expression for *miR-21*, *miR-146b*, *miR-221*, *miR-222*, *miR-31*, *miR-3613*, *miR-138*, and *miR-98*. Moreover, this study also sought to determine whether there were noticeable differences in aberrant miRNA expression in tumor specimens between CA and AA patients. Based on the sample size limitation, this study does not claim to provide definitive conclusion on differential expression of miRNAs between the two racial groups. However, it does aim to draw scientific attention to the possibility of explaining differences in the risk for PTC mortality between CA and AA patients, which may be partly contributed by differential expression of miRNAs. Our results may facilitate the determination of which pathways are most important for future development of miRNA-targeted therapies to optimize personalized treatments of PTC patients.

Overall, we found 6 miRNAs that were up-regulated and 2 miRNAs that were down-regulated in PTC tumors. Up-regulated miRNAs included *miR-21*, *miR-146b*, *miR-221*, *miR-222*, *miR-31*, and *miR-3613*. Down-regulated miRNAs were *miR-138* and *miR-98*. We found that *miR-221* was significantly up-regulated to a greater extent in CA than in AA. We also found that *miR-31* appears to be up-regulated in CA, whereas it appears to be down-regulated in AA. Moreover, we found up-regulation of *miR-31* in PTC tumors, although *miR-31* has been found to be down-regulated in other types of cancer, including prostate cancer and esophageal cancer [19,20]. Improved understanding on the mechanistic role of *miR-31* and *miR-221* in PTC and their role in defining racial disparity could prove to be useful in optimizing tailored therapeutic strategies in the future.

## Materials and Methods

### Tissue collection

Histopathology slides from papillary thyroid cancer patients were microscopically reviewed by two pathologists (SS and TG). Representative blocks were selected that contained >70% tumor cells for deciding which sections of the tumor to be used when collecting tumor samples. For normal and tumor tissue sections, five sections, each of 10 microns in thickness, were cut from the selected blocks and placed into sterile Eppendorf tubes.

### RNA isolation

The RNA was isolated from FFPE tissues using the RNeasy Kit (Qiagen, Valencia, CA, USA) in accordance with the manufacturer’s protocol. Briefly, tissue sections were placed in micro-tubes, and 1 ml xylene was added. After vigorous shaking for 10s, samples were centrifuged at 13,000 × g for 2 min at room temperature. The supernatant was removed, and the pellet was re-suspended in 240 μl of Buffer PKD along with 10 μl of Proteinase K as described previously [21]. RNA was washed with buffer solution to remove impurities and eluted in a final volume of 15 μl. RNA was quantified and its purity was evaluated by the absorption ratio at 260/280 nm using NanoDrop 2000 (Thermo Scientific, Pittsburgh, PA, USA).

### MicroRNA profiling

Extracted RNA was pooled into four tubes such as: CA normal and CA tumor; and AA normal and AA tumor. LC Sciences then quantitatively analyzed the RNA (tested both the quantity and the quality prior to array) for miRNA microarray profiling, using miRBase version 18 (LC Sciences, Houston, TX, USA). Selected housekeeping genes were used to normalize the data. Furthermore, the web-based Ingenuity pathway analysis software was used to perform network analysis (Ingenuity Systems, Redwood City, CA, USA).

### Quantitative real-time polymerase chain reaction (qRT-PCR)

Quantitative-RT-PCR was performed on the individual samples in order to validate the miRNA profiling results of 8 selected miRNAs using TaqMan Universal PCR Master Mix, no AmpErase UNG. Selected miRNAs were *miR-21*, *miR-146b*, *miR-221*, *miR-222*, *miR-3613*, *miR-31*, *miR-138*, and *miR-98*. Though 14 CA patients were studied for some miRNAs, the tissue sample was insufficient for one CA patient. Thus, the other remaining miRNAs were studied in 13 patients whose tissue sample sizes were adequate. There was an insufficient amount of tissue samples for 3 AA patients. Thus, some miRNAs were studied in 8 AA patients, and the remaining miRNAs were studied in 5 AA patients. The High Capacity cDNA Reverse Transcription Kit (Applied BioSystems, Foster City, CA, USA) was used per manufacturer’s protocol. Approximately 10 ng of RNA from the respective tissue specimens was reverse transcribed using 7 μl of master mix and 3 μl of RT primer. The mixture was incubated at 16°C for 30 min, then 42°C for another 30 min, and finally 85°C for 5 min. Reverse transcriptase (RT)-PCR reactions were then carried out in triplicate with 2.26 μl of RT product mixed with 1.7 μl of probe and 26 μl of TaqMan master mix. Each of the three wells had a volume of 10 μl. All reactions, including controls, were performed using StepOnePlus Real-Time PCR (Applied BioSystems, Foster City, CA, USA). Relative expression of miRNAs was analyzed using the Ct method and was normalized by RNU48 expression.

### Statistical analysis

Differences in the expression levels of miRNAs between groups were statistically evaluated by using the F test to compare variances using GraphPad StatMate software (GraphPad Software Inc.). Variances that were found to be significantly different had a p value of less than 0.001.

## Results

### The profiling of miRNA expression

**Figure 1:**
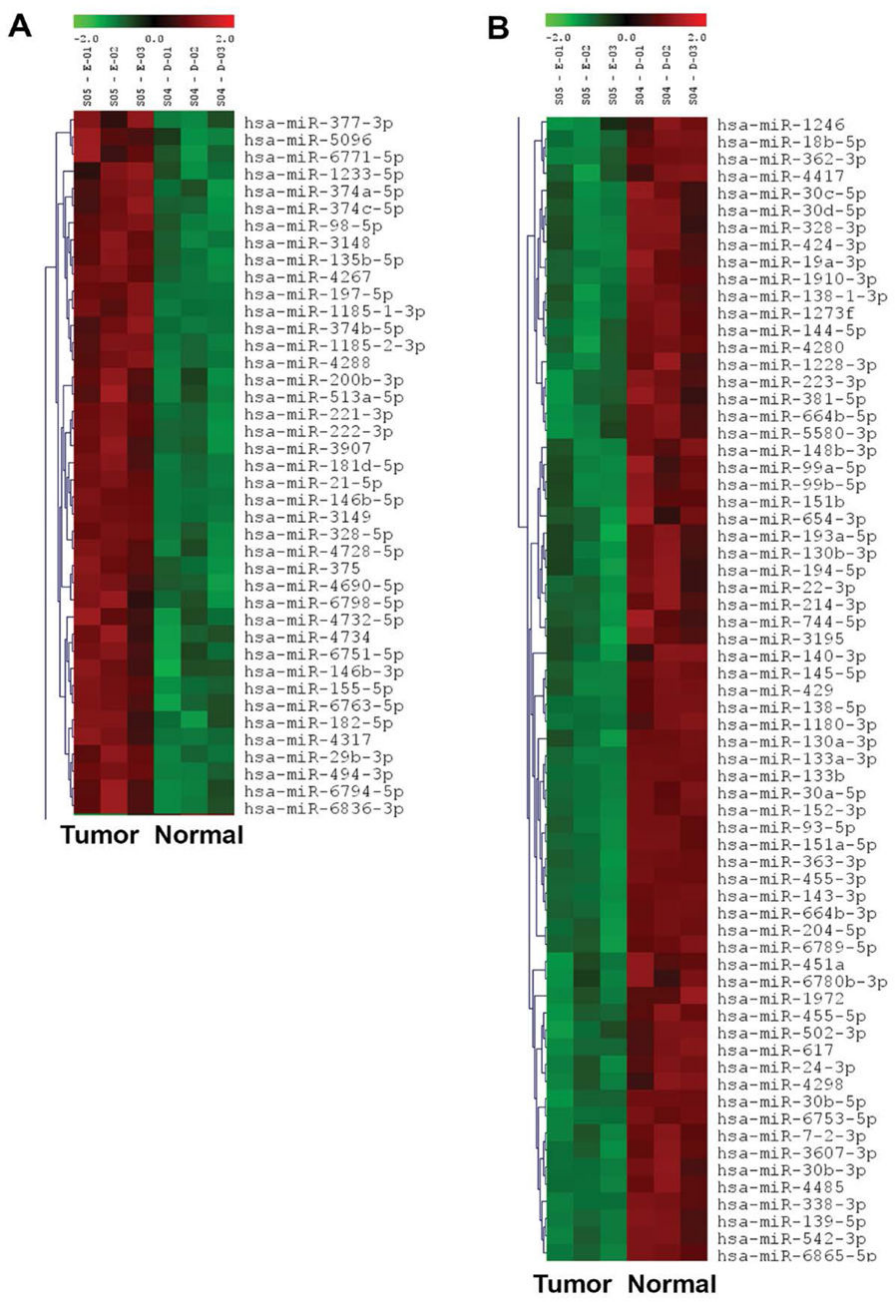
A hierarchical clustering and heat maps of the miRNA profiling of Caucasian American (normal vs tumor samples), up-regulated in tumor samples (A) and down-regulated in tumor samples (B).

Expression profiling revealed 107 miRNAs that were differentially expressed between normal CA tissue and tumor CA tissue samples. Furthermore, 66 miRNAs were differentially expressed between normal AA tissue and tumor AA tissue samples. The data shown in Figure 1 is representative heat map of CA tissue samples compared between normal and tumor tissue, red indicates up-regulation and green indicates down-regulation of miRNAs. It was interesting to observe the differential expression of more miRNAs in CA than in AA. However, it is premature to overestimate the significance of this difference. It would take patient-by-patient validation of each miRNA to ensure that no one patient was artificially inflating any differences in miRNA levels between normal and tumor, which was beyond the scope of the current investigation. Based on the above results of miRNA profiling, we chose eight miRNAs that were found to be significantly deregulated between normal and tumor samples for further validation using qRT-PCR. These miRNAs included *miR-21*, *miR-146b*, *miR-221*, *miR-222*, *miR-31*, *miR-3613*, *miR-138* and *miR-98*. The analyses of our findings on these eight miRNAs are described in the following sections.

### Pathway Analysis for miRNAs

**Figure 2:**
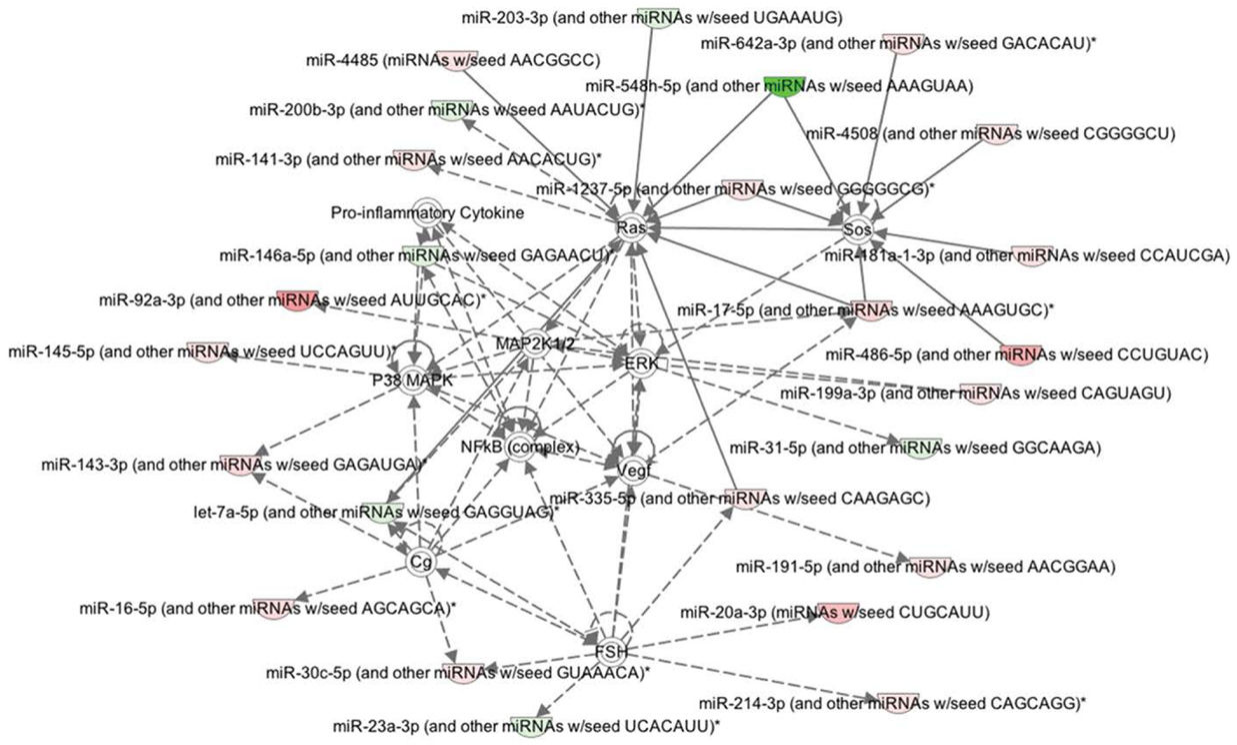
Ingenuity pathway analysis showing up (green) and down-regulation (red) of miRNAs involved in papillary thyroid cancer (PTC) tumor samples when compared to normal samples. Target genes are also represented, such as RAS, NF-κB, VEGF, and P38 MAPK pathways.

In an attempt to understand the target genes and pathways that are involved in PTC, Ingenuity modeling of the miRNA profiling was conducted. Specific networks were algorithmically generated based on their connectivity. The analysis revealed the influence of many commonly studied pathways such as Ras, NF-κB, MAP kinase, and VEGF, as depicted in Figure 2.

### Quantitative Real-Time PCR of selected miRNAs

**Figure 3:**
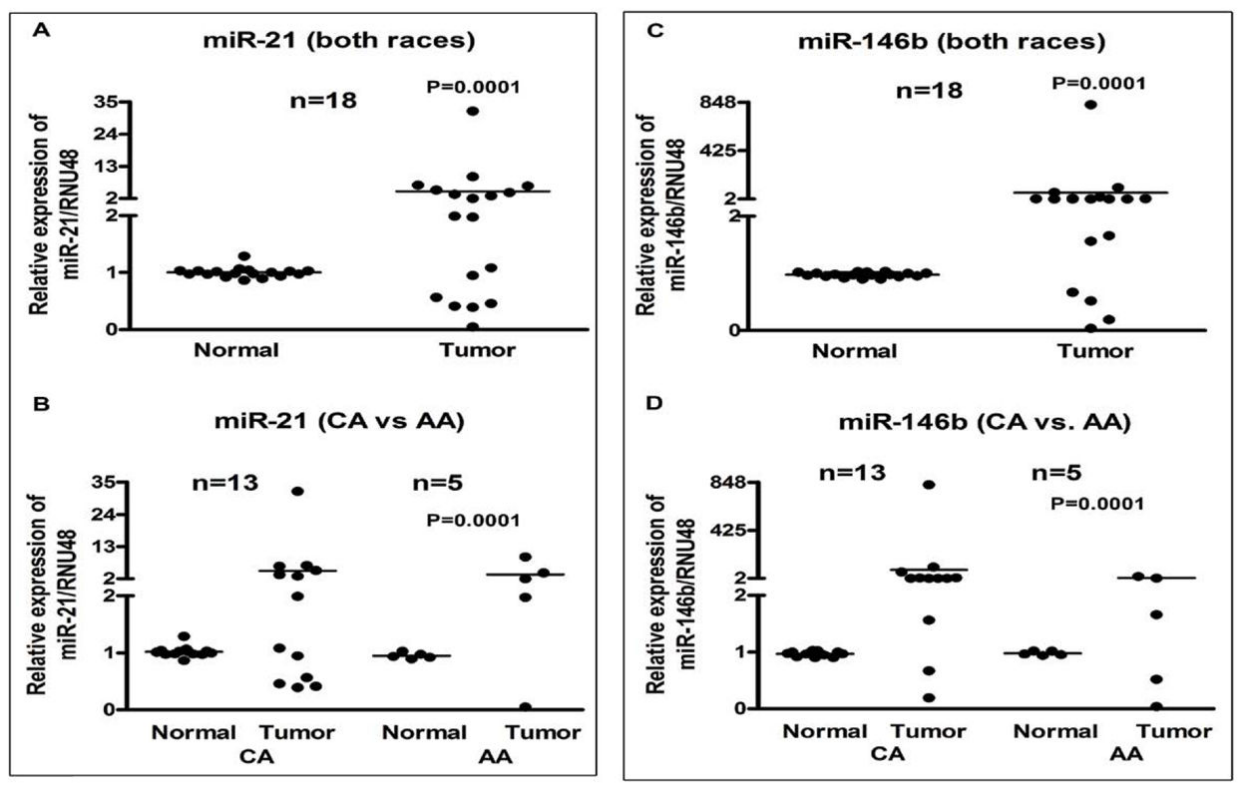
Comparative expression analysis of *miR-21* and *miR-146b* in 18 paired samples of FFPE cell blocks of tumor and normal tissue from papillary thyroid carcinoma (PTC) patients’ individually using qRT-PCR. Combined data for both races (A and C) and race-specific data (B and D) is presented. There was a significant up-regulation of *miRNA-21* in African American (AA) patients when compared to Caucasian American (CA) patients (3B). Overall, while *miR-146b* appears to be up-regulated, a larger percentage of CA samples than AA samples followed this trend (3D). The miRNAs expression was normalized using RNU48 miRNA. *P values* represent comparison between normal and tumor (3A, 3C), and CA tumor vs AA tumor (3B, 3D) using F test.

**Figure 4:**
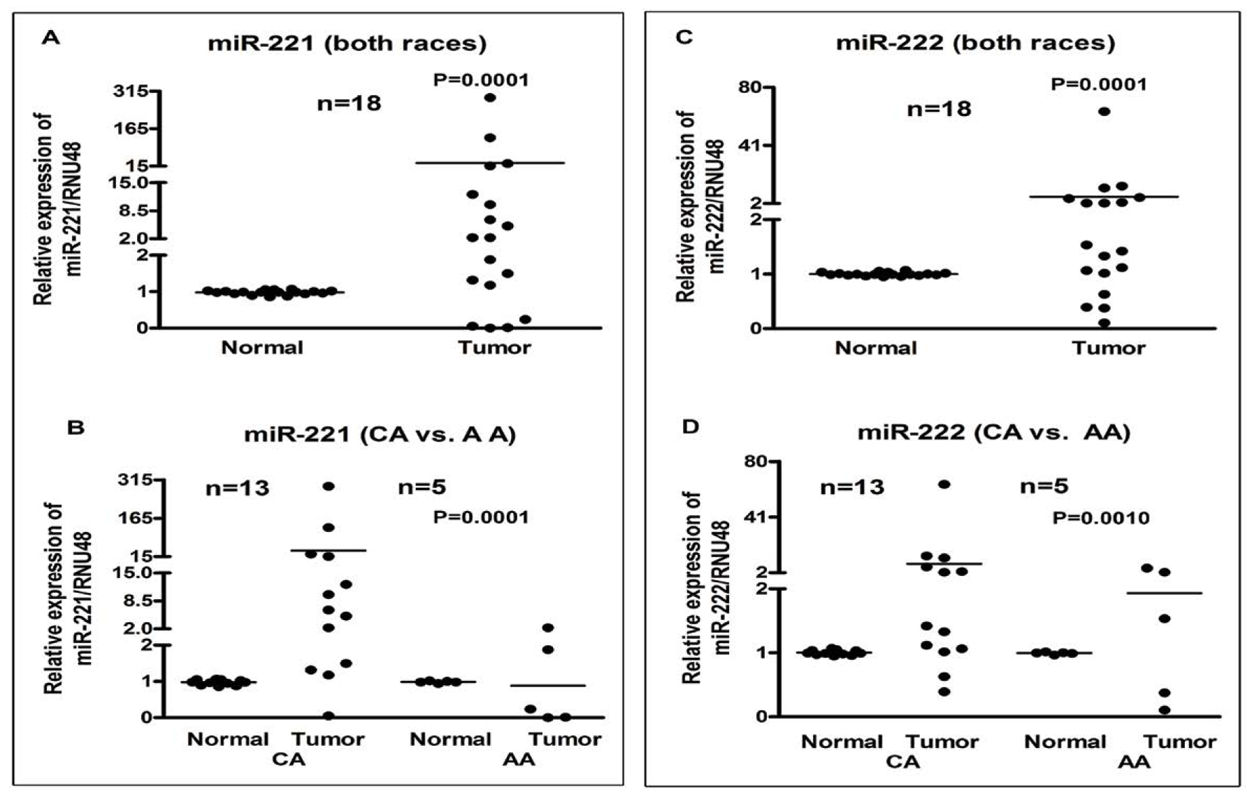
Comparative expression analysis of *miR-221* and *miR-222* in 18 paired samples of FFPE cell blocks of tumor and normal tissue from PTC patients’ individually using qRT-PCR. Combined data for both races (A and C) and race-specific data (B and D) is presented. For *miR-221*, there appears to be greater up-regulation in CA tumors than in AA tumors (4B). No such race-specific difference was observed for *miR-222*. The miRNAs expression was normalized using RNU48 miRNA. *P values* represent comparison between normal and tumor (4A, 4C), and CA tumor vs AA tumor (4B, 4D) using F test.

**Figure 5:**
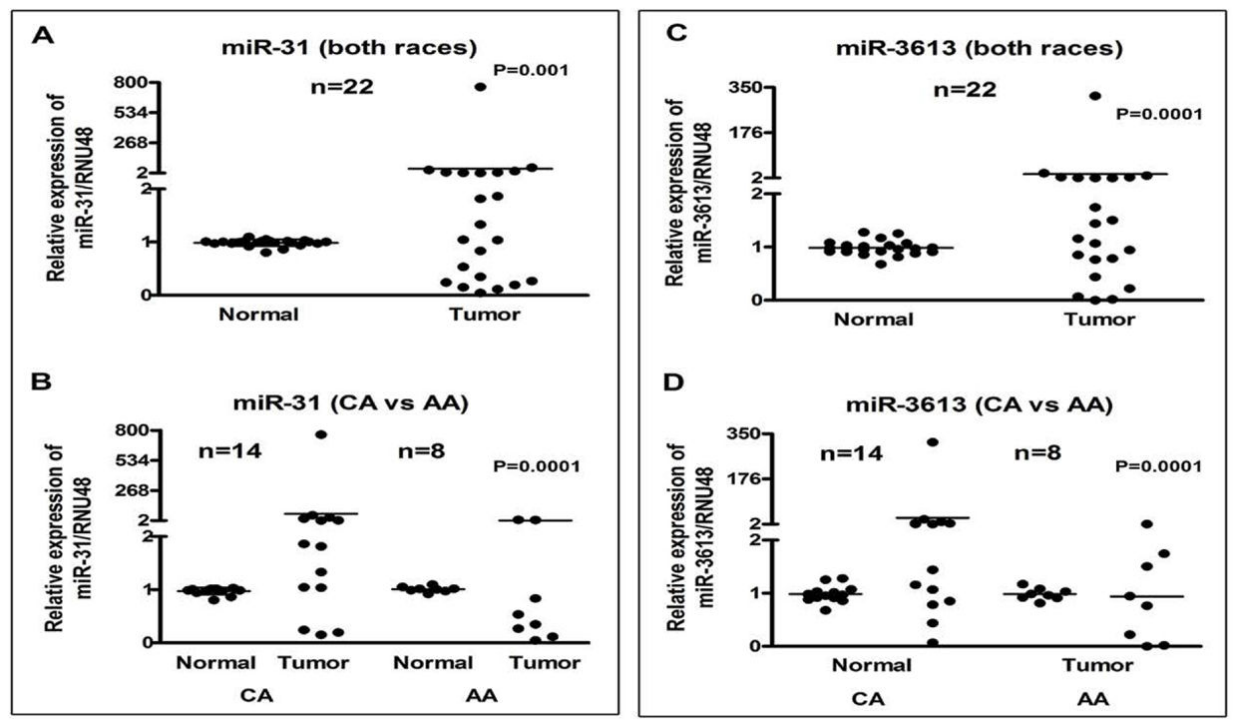
Comparative expression analysis of *miR-31* and *miR-3613* in 22 paired samples of FFPE cell blocks of tumor and normal tissue in PTC patients’ individually using qRT-PCR. Combined data for both races (A and C) and race-specific data (B and D) is presented. In combined data for *miR-31*, there appears to be no trend regarding differential miRNA expression (5A). When individual races were examined, *miR-31* was mostly up-regulated in CA patients and primarily down-regulated in AA patients (5B). No such race-specific difference was observed in *miR-3613*. The miRNAs expression was normalized using RNU48 miRNA. *P values* represent comparison between normal and tumor (5A, 5C), and CA tumor vs AA tumor (5B, 5C) using F test.

Based on the miRNA profiling data, 8 miRNAs were chosen for validation in individual samples obtained from CA and AA patients. Paired samples of normal and tumor tissue from 14 CA and 8 AA were analyzed where ever possible. The characteristics of the 22 patients showing age, gender, race, and tumor size, etc. are presented in Table 1. Upon discovery of insufficient sample size, remaining analyses were conducted on paired samples of 13 CA and 5 AA. Analyses were performed in parallel in order to avoid batch effects. The miRNA expression analysis showed that 6 miRNAs were mostly up-regulated in tumor compared to the normal tissue. These miRNAs were *miR-21*, *miR-146b* (Figure 3), *miR-221*, *miR-222* (Figure 4), *miR-31*, and *miR-3613* (Figure 5). These miRNAs showed significant up-regulation in most of the tumors compared to normal controls. Expression levels were compared between all normal and all tumor tissues of both races, as seen in Figure 3A, and in individual racial groups, as seen in Figure 3B. This was done to determine if overall trends in race-specific differences could be seen. As observed in Figure 3B, though the sample size was small, AA showed significant overexpression of *miR-21* in all except one patient. Thus, 80% of AA tumors showed *miR-21* up-regulation. On the other hand, *miR-21* expression in CA showed up-regulation in about 50% of tumors.

Conversely, *miR-146b* showed overall up-regulation when considering both races together (Figure 3C). Upon closer inspection, 85% of CA tumors showed up-regulation (Figure 3D) while only 60% showed *miR-146b* up-regulation in AA patients.

When *miR-221* was examined in both races together, there appeared to be an overall up-regulation (Figure 4A). Of the CA tumors, 92% showed *miR-221* up-regulation. On the other hand, only two AA tumors showed up-regulation, while the remaining three were down-regulated (Figure 4B). We further assessed the expression of *miR-222* appeared to be overall up-regulated in tumors (Figure 4C). When analyzing for race-specific differences, there were no overwhelming differences in *miR-222* expression between CA and AA patients (Figure 4D).

There was no trend of differential expression in the expression of *miR-31* between normal and tumor (Figure 5A). Half of the tumors were up-regulated and the other half were down-regulated. Upon consideration of race-specific information, it seemed that *miR-31* was largely up-regulated in CA tumors, while it was down-regulated in 6 out of 8 AA tumors (Figure 5B).

The expression of *miR-3613* appeared to be up-regulated when considering both races together (Figure 5C). In the tumors examined, 77% showed either an up-regulation or equivalent level of expression when compared to normal tissue (Figure 5D). There was no race-specific difference in the differential expression of *miR-3613* in the samples studied.

**Figure 6:**
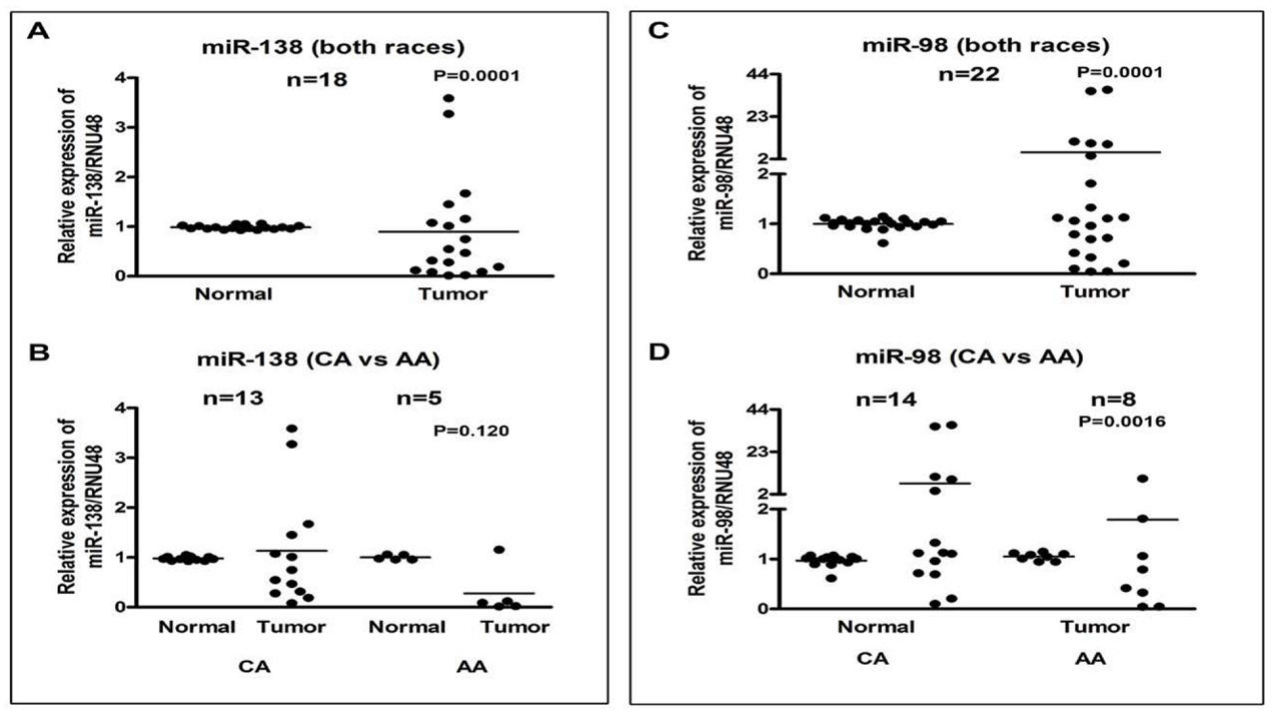
Comparative expression analysis of *miR-138* in 18 paired samples and *miR-98* in 22 paired samples of FFPE cell blocks of tumor and normal tissue from PTC patients’ individually using qRT-PCR. Combined data for both races (A and C) and race-specific data (B and D) is presented. No clear trend was present for *miR-98* expression in CA. However, in AA, 80% of tumors had down-regulated *miR-138* (Figure 6B). The miRNAs expression was normalized using RNU48 miRNA. *P values* represent comparison between normal and tumor (6A, 6C), and CA tumor vs AA tumor (6B, 6D) using F test.

Furthermore, there were two miRNAs that were largely down-regulated. These miRNAs were *miR-138* and *miR-98* (Figure 6). There was an overall trend of down-regulation of *miR-138* in tumors (Figure 6A). In CA, 50% of the tumors showed *miR-38* down-regulation whereas 80% of AA tumors had down-regulated expression of *miR-138* (Figure 6B). Finally, *miR-98* was found to be down-regulated in about half of the samples studied (Figure 6C). This finding was consistent even when considering both races individually. The *miR-98* was still considered a down-regulated miRNA because in only a very few tumors it was up-regulated; the rest were either down-regulated or had constant levels of expression in both normal and tumor tissues.

## Discussion

Understanding the role miRNAs in papillary thyroid cancer (PTC) is crucial for thorough molecular understanding of this disease. The expression of miRNAs has previously shown clinical significance in the diagnosis of PTC [22]. Furthermore, miRNAs have been used as predictive biomarkers for cancer prognosis [23]. It is anticipated that increasing the body of knowledge with regard to the differential expression of miRNAs in PTC would be useful for further development of novel tailored therapies in order to improve the treatment outcome of PTC patients. In addition, if the expression of miRNAs varies between the two racial groups then this race-specific knowledge could be invaluable for the development of tailored miRNA-targeted therapies. Finally, since miRNAs are so small when compared to RNAs, only samples of smaller sizes are required for research investigation, making miRNAs very practical to work within the laboratory for assessing their clinical significance. Though miRNAs have proven to be extremely useful in the study and treatment of cancer in recent years, it is important to remember the potential impact of tumor heterogeneity in reaching final conclusion. Thus, discussion of our results is solely based on overall trends that we observed during our data analysis and the concept of tumor heterogeneity has not been addressed in this study.

Of the eight miRNAs that we chose for qRT-PCR validation, two appear to be down-regulated in tumor tissue. One of these was *miR-138*, an anti-oncogenic miRNA. In human anaplastic thyroid carcinoma, *miR-138* down-regulation was shown to be concomitant with overexpression of human telomerase reverse transcriptase protein, or hTERT [24]. The human hTERT is responsible for allowing DNA to replicate excessively while preventing telomere erosion [25]. In normally functioning cells, *miR-138* works with other miRNAs to inhibit telomerase activity. When these telomerase-inhibiting miRNAs are down-regulated, heightened telomerase activity provides the opportunity for uncontrolled replication, and thus the onset of tumorigenesis. Therapeutic miRNA delivery of *miR-138* with other down-regulated miRNAs could prove helpful for PTC patients, as this would reduce telomerase functioning.

Additionally, the majority of patients appeared to have either down-regulation of *miR-98* in tumor cells or no change in its expression. The *miR-98* has not been extensively studied in PTC. One study examining *miR-98* expression in breast cancer determined that *miR-98* was down-regulated in metastatic tumors when compared to normal tissues [26]. The same study found that transfection of a breast cancer cell line with pre-*miR-98* resulted in increased apoptosis, as observed by fluorescence-activated cell sorting (FACS). When normally expressed, *miR-98* may down-regulate Activin A receptor, type IB (ALK4) and Matrix-metalloproteinase-11 (MMP11). In the absence of *miR-98*, ALK4 and MMP11 expression are increased. Thus, the down-regulation of *miR-98* may contribute to PTC through similar mechanisms. In fact, two studies found increased MMP11 in PTC when compared to normal tissue [27,28], which is consistent with down-regulation of *miR-98* expression. However, one study showed that there was decreased expression of ALK4 in PTC compared to normal thyroids. We encourage researchers to look into the role of *miR-98* and its possible mechanisms of action in PTC. It is tempting to speculate that there may be a potential mechanistic link related to the molecular action of *miR-98* between breast cancer and thyroid cancer as suggested by an observation showing increased risk of thyroid cancer in young breast cancer survivors [29].

Other miRNAs, including *miR-21*, were up-regulated in PTC. The observed up-regulation of *miR-21* is consistent with the findings reported by others on miRNA-based PTC research [6,12]. Studies have even gone on to propose the downstream effects of *miR-21* up-regulation. One study proposed that the effect of aberrant *miR-21* expression led to the suppression of a tumor suppressor gene, programmed cell death 4, or PDCD4. This PDCD4 targeting could result in enhanced proliferation and reduced apoptosis of PTC cells [6]. Previous research has demonstrated that another target gene of *miR-21* is thyroid hormone receptor beta (THRB) which is an important tumor suppressor gene [12]. In essence, the role of *miR-21* in PTC is the suppression of known tumor suppressors, which is consistent with other cancers. Therefore the effects of *miR-21* knockdown should be studied in PTC in future studies, especially because the knockdown of *miR-21* in human breast cancer cell lines has been found to inhibit tumor growth *in vivo* [30].

Furthermore, *miR-146b* was found to be up-regulated in CA patients, which is consistent with published studies [8,13,31]. The *miR-146b* has been found to promote the migration and invasiveness of PTC cells. Additionally, it promoted the transition of epithelial cells to epithelial-mesenchymal transition (EMT) of PTC cells [13]. One of the proposed targets of *miR-146b* is ZNRF3, a tumor suppressor gene [13]. Interestingly, the same study showed that the overexpression of ZNRF3 reversed the migration, invasiveness, and EMT effects of *miR-146b* on PTC cells.

Additionally, we examined *miR-221* expression. Though *miR-221* was largely up-regulated in CA patients, it was significantly down-regulated in three out of five AA patients studied, and thus the significance of such finding appears to be provocative. When studying NSCLC, Heegaard et al. reported that AA patients show decreased expression of both *miR-146b* and *miR-221* in the plasma when compared to CA patients [32]. This observation is consistent with our findings, as we found that *miR-221* was more up-regulated in CA patients. Both previous literature and the results of our study suggest that *miR-221* appears to be up-regulated in PTC in CA [5,9,33]. This has already had some potential therapeutic implications. One study determined that the blocking of *miR-221* function through the use of anti-sense methodology resulted in reduced cell growth of a PTC cell line [5]. In breast cancer, *miR-221* knockdown reversed EMT and weakened cancer cells’ ability to migrate and invade [34]. Further research needs to be done, but optimistically, *miR-221* knockdown would have similar effects in PTC, at least, in CA patients. Interestingly, *miR-221* overexpression could be an early event in carcinogenesis, as postulated by another study showing that *miR-221* up-regulation in normal thyroid tissue [9]. This explanation could also provide some insight as to why three of the five AA tumors showed down-regulation of *miR-221* in tumor cells. However, if further studies established *miR-221* as consistently more up-regulated in CA patients, then this could provide an insight into race-specific differences in the expression of *miR-221* in the biology of PTC.

Another up-regulated miRNA was *miR-222*, which showed increased expression in PTC tumors regardless of race. When Pallante et al. studied PTC using a miRNA-CHIP microarray; they also noticed an up-regulation of *miR-222*, which they confirmed through Northern blot analysis as well as qRT-PCR [5]. They postulated that *miR-222* up-regulation was a signature of human PTC. When other research looked more closely into *miR-222* overexpression, it was determined that its up-regulation occurred concomitantly with a significant loss of KIT transcript and KIT protein [4]. This overall effect was determined to contribute to thyroid pathogenesis.

The expression of *miR-31* also appeared to be up-regulated in CA tumors. When compared to other miRNAs, *miR-31* has not been extensively studied in PTC. Tetzlaff et al. found up-regulation of *miR-31* expression in PTC [14]. However, they compared the expression levels in PTC tumors to those in multi-nodular goiter [14]. Compared to other miRNAs, *miR-31* has been less extensively studied in PTC as indicated earlier. In our study, a striking finding was that *miR-31* appears to be up-regulated in tumors from CA patients and down-regulated in tumors from AA patients. As demonstrated in Figure 2, *miR-31* appears to be connected with upstream mitogen-activated protein kinase/extracellular regulated kinase (MAPK/ERK) signaling. Thus, increased MAPK/ERK activity could be causatively linked with deregulated expression of *miR-31* in CA patients. Consistent with our findings, another study demonstrated that rat *miR-31* up-regulation in vascular smooth muscle cells was inhibited by the inhibitor of MAPK/ERK [35]. The down-regulation of the MAPK pathway has been previously postulated to play an important role in colorectal cancer [36]. Investigators who measured levels of phosphorylated protein isoforms in colorectal cancer discovered that there was a two to four-fold decrease in activated signaling molecules (such as p-ERK, p-p38, and p-JNK) in cancers when compared to uninvolved mucosa [36]. However, it is important to consider that *miR-31* like many other miRNAs have multiple targets which are deregulated in a context dependent manner.

Furthermore, the aberrant expression of protein p21, a cyclin-dependent kinase inhibitor, has been implicated in cancer [37]. In specific cellular environments *miR-31* has shown tumor-suppressive activities in esophageal carcinoma and liver cancer [38,39]. In esophageal cancer, *miR-31* only exhibited tumor suppressive activities when p21 levels were low [39]. The overexpression of p21 protein has been proposed as an indicator of poor prognosis in papillary thyroid cancer [40]. This could be related to the resultant inability of *miR-31* to engage in its tumor suppressive functions. African American papillary thyroid tumor could have low levels of p21, leading to the potential utility of *miR-31* in tumor suppression. Thus, cancer cells would have developed mechanisms to silence tumor suppressive *miR-31* in the African American p21-low tumor cells, which in part may be mediated through, the down-regulation of the MAPK pathway. Our preliminary findings warrants further future research to examine both race-specific *miR-31* differences and p21 expression levels in a larger patient population.

The last miRNA we examined was *miR-3613*. One study examining miRNA expression in colon cancer found *miR-3613* enrichment in an extracellular vesicle subtype [41]. Since extracellular vesicles provide a mechanism for the secretion of miRNAs, this enrichment was noteworthy. However, the downstream effects of *miR-3613* up-regulation have not been established or even explored to-date.

Although our study had a small sample size, we found a wide range of variation concerning differential miRNA expression in PTC. Previous research has demonstrated that certain miRNAs are differentially expressed by tumor size [42]. In fact, one study examining circulating miRNA expression in the plasma of PTC patients determined that the levels of *miR-146b* and *miR-222* increased in proportion to tumor size [31]. This could begin to explain the spread of values seen in graphical representations of the relative expressions of studied miRNAs. Future studies attempting to examine miRNA differences between races should include tumors of similar sizes. Further explanations for patient-specific differences in the relative increase or decrease in the expression of miRNAs could stem from the characteristics of PTC present in patients. For example, patients presenting with capsule invasion, vascular invasion, or lymph node metastasis were found to have higher expressions of *miR-146b*, *miR-221*, and *miR-222* [33]. This could account for why certain patients exhibited much higher up-regulation of these miRNAs than others. Additionally, Yip et al. observed differential expressions of *miR-31*, *miR-146b*, *miR-221*, *miR-222*, and *miR-138* between aggressive and non-aggressive PTC [10]. The expression of *miR-146b*, *miR-221*, and *miR-222* were up-regulated in aggressive PTC, and *miR-138* was down-regulated in aggressive PTC [10]. Thus, the varying aggressiveness of the tumors could have had some impact on the wide spread of our data as presented in Figures 3–6.

In summary, we envision that tissue-based miRNA research will have a powerful clinical implication in thyroid cancer. Working towards understanding the molecular mechanisms of PTC will naturally provide tools for the development of miRNA-targeted personalized therapy for PTC.

## Figures and Tables

**Table 1:** Characteristics of the 22 papillary thyroid carcinoma patients at time of diagnosis.

Patient #	Gender	Age (Year)	Race	Tumor Size (cm)	MET/Non-MET
31	F	66	CA	0.5	Non-MET
32	F	38	CA	0.2	Non-MET
33	F	52	CA	1.2	Non-MET
34	F	29	CA	1.9	MET
35	F	45	CA	0.1	Non-MET
36	F	44	CA	0.9	Non-MET
37	F	53	CA	3.8	MET
38	M	77	CA	2.5	MET
39	F	53	CA	0.1	Non-MET
40	F	39	CA	0.1	Non-MET
41	F	77	CA	1.2	MET
42	F	29	CA	5.5	MET
43	M	57	CA	1.0	MET
44	F	43	CA	0.7	Non-MET
63	F	44	AA	1.5	Non-MET
64	F	33	AA	4.5	MET
65	M	24	AA	2.0	Non-MET
66	F	28	AA	1.5	MET
67	F	52	AA	3.0	MET
68	M	47	AA	0.2	Non-MET
69	F	64	AA	0.2	Non-MET
70	F	45	AA	1.3	Non-MET

Tumor size refers to the diameter of the largest tumor in the thyroid gland.

MET: Metastases, Non-MET: Non-Metastases
